# A comparative study on characteristics of composite (Cr3C2-NiCr) clad developed through diode laser and microwave energy

**DOI:** 10.1038/s41598-023-37991-4

**Published:** 2023-07-04

**Authors:** Ajit M. Hebbale, Manish Kumar, Manzoore Elahi Mohammad Soudagar, Tansir Ahamad, Md. Abul Kalam, Nabisab Mujawar Mubarak, Akram Alfantazi, Mohammad Khalid

**Affiliations:** 1 Department of Mechanical Engineering, Nitte (Deemed to Be University), NMAM Institute of Technology (NMAMIT), Nitte, Karnataka 574110 India; 2 Department of Mechanical Engineering and University Centre for Research & Development, Chandigarh University, Mohali, Punjab 140413 India; 3 Institute of Sustainable Energy, Universiti Tenaga Nasional, 43000 Kajang, Selangor Malaysia; 4 Department of Chemistry, College of Science, King Saud University, Riyadh, Saudi Arabia; 5 School of Civil and Environmental Engineering, FEIT, University of Technology, Sydney, NSW 2007 Australia; 6 Petroleum and Chemical Engineering, Faculty of Engineering, Universiti Teknologi Brunei, Bandar Seri Begawan, 1410 Brunei Darussalam; 7 Department of Chemical Engineering, Khalifa University, 127788 Abu Dhabi, United Arab Emirates; 8 Sunway Centre for Electrochemical Energy and Sustainable Technology (SCEEST), School of Engineering and Technology, Sunway University, No. 5 Jalan Universiti, Bandar Sunway, 47500 Petaling Jaya, Selangor, Malaysia; 9 Division of Research and Development, Lovely Professional University, Phagwara, Punjab 144411 India

**Keywords:** Energy science and technology, Engineering, Materials science, Nanoscience and technology

## Abstract

A typical ferrite/martensitic heat-resistant steel (T91) is widely used in reheaters, superheaters and power stations. Cr_3_C_2_-NiCr-based composite coatings are known for wear-resistant coatings at elevated temperature applications. The current work compares the microstructural studies of 75 wt% Cr_3_C_2_- 25 wt% NiCr-based composite clads developed through laser and microwave energy on a T91 steel substrate. The developed clads of both processes were characterized through a field emission scanning electron microscope (FE-SEM) attached with energy-dispersive X-ray spectroscopy (EDS), X-ray diffraction (XRD) and assessment of Vickers microhardness. The Cr_3_C_2_-NiCr based clads of both processes revealed better metallurgical bonding with the chosen substrate. The microstructure of the developed laser clad shows a distinctive dense solidified structure, with a rich Ni phase occupying interdendritic spaces. In the case of microwave clad, the hard chromium carbide particles consistently dispersed within the soft nickel matrix. EDS study evidenced that the cell boundaries are lined with chromium where Fe and Ni were found inside the cells. The X-ray phase analysis of both the processes evidenced the common presence of phases like chromium carbides (Cr_7_C_3,_ Cr_3_C_2_, Cr_23_C_6_), Iron Nickel (FeNi_3_) and chromium-nickel (Cr_3_Ni_2_, CrNi), despite these phases iron carbides (Fe_7_C_3_) are observed in the developed microwave clads. The homogeneous distributions of such carbides in the developed clad structure of both processes indicated higher hardness. The typical microhardness of the laser-clad (1142 ± 65HV) was about 22% higher than the microwave clad (940 ± 42 HV). Using a ball-on-plate test, the study analyzed microwave and laser-clad samples' wear behavior. Laser-cladding samples showed superior wear resistance due to hard carbide elements. At the same time, microwave-clad samples experienced more surface damage and material loss due to micro-cutting, loosening, and fatigue-induced fracture.

## Introduction

Surface modification techniques are critical in improving the performance and durability of engineering components subjected to severe wear and corrosion. Because of its high wear resistance and corrosion, composite cladding, notably the Cr_3_C_2_-NiCr system, has received much attention. However, the energy source used in the cladding process significantly impacts the clads' final properties and overall performance^1^. Ceramic/metal composite materials, such as cermets, have long been recognized as a prominent solution for enhancing mechanical components' wear and corrosion resistance in industrial applications. However, conventional approaches like ingot or powder metallurgy present significant challenges in producing cermet composites. Alternatively, surface engineering technologies like thermal spraying, laser cladding, and microwave cladding offer practical approaches to developing functional coatings on industrial components, effectively safeguarding target surfaces from wear and corrosion-related issues^2,3^. Among these techniques, High-Velocity Oxygen Fuel (HVOF) spraying is a commercially viable choice for creating various cermet coatings. The coatings developed through the HVOF process exhibit substantial bonding strength with minimal porosity. However, eliminating coating pores during HVOF coating is challenging, leading to lower bonding strength than metallurgical bonding^4^. These drawbacks significantly limit the industrial applications of the HVOF process, as the presence of pores within the coatings can serve as accelerated diffusion paths for corrosive environments, posing a critical threat to the component's service life^5,6^.

The laser cladding process presents an alternative technique for coating applications, offering precise control over dilution and metallurgical bonding, which facilitates the development of refined microstructures. This technique boasts various advantages, including low porosity with a fully dense structure, minimal damage to the target substrate at the interface, and robust metallurgical bonding. The laser cladding process has recently gained significant attention in high-temperature wear-resistant coatings, making it a prominent topic in material surface modification. For instance, Jayaprakash et al.^7^ examine the characteristics of laser alloyed WC-12%Co and Cr_3_C_2_-25%NiCr powders over nodular cast iron and their outcomes on microstructure, microhardness, and wear resistance properties. The contribution of this paper is to deliver insights into the microstructure and tribological evolution during laser alloying of WC-12%Co and Cr_3_C_2_-25%NiCr powders on nodular iron surfaces, which can be beneficial for developing wear-resistant coatings for industrial applications. Another study reported that the laser cladding of NiCr/Cr_3_C_2_-30%WS_2_ composite coating can effectively minimize friction and wear characteristics at temperatures up to 3000 °C^8^. Laser remelting of thermally sprayed coatings has also been extensively studied for various material systems, such as Ni-based self-flowing alloys, WC–Co, or Cr_3_C_2_-NiCr cermets, using in situ laser irradiation. It was observed that the depth of melting increases with higher input laser energy density^5,9^.

Additionally, Chenggang et al.^10^ investigated the laser cladding of Ni60-Cr_3_C_2_ powder on alloy W_6_Mo_5_Cr_4_V_2_, revealing a significant increase in hardness attributed to the formation of a solid solution of Cr_3_C_7_ carbides. However, it is important to note that the laser cladding process has limitations, including high thermal stress due to the steep thermal gradient, residual stress, and porosity. These limitations necessitate innovative cladding methods to overcome these challenges, as laser coatings are cost-effective for processing large surfaces and require solutions to address these issues.

The increasing demand for energy efficiency, time effectiveness, and environmentally friendly approaches has driven the development of novel surface modification techniques to meet global industry standards. In recent years, microwave energy utilization for material processing has emerged as a promising avenue that aligns with these requirements, offering an alternative to eco-friendly and energy-efficient methods^11^. The microwave cladding process has experienced significant growth in recent times. This technique leverages the volumetric heating nature of microwaves, which ensures a uniform thermal gradient throughout the clad structure and facilitates superior metallurgical bonding with the substrate. Moreover, microwave cladding is characterized by its energy efficiency, cost-effectiveness, and reduced processing time. Initial studies by Guptha and Sharma^12^ have explored the application of microwave energy for cladding metallic materials onto stainless steel. Subsequently, research by various authors has demonstrated extensive work on the microwave cladding process. The developed microwave clads exhibited enhanced metallurgical bonding with the substrate, devoid of cracks or pore formations^12–16^. Furthermore, positive results have been achieved in producing Ni-SiC, Ni-Cr_3_C_2_, and Ni-WC-based composite clads using microwave energy on different grades of stainless steel^17,18^.

In modern industry, hard chromium coatings are widespread to improve the wear performance of engineering components like pistons and valves. However, due to the detrimental environmental effects and health hazards associated with chromium compounds, there have been numerous efforts to find alternative coatings^19^. Additionally, conventional hard chromium coatings experience a drop in mechanical properties at temperatures above 350 °C. As a result, seeking alternatives such as coatings containing Cr_3_C_2_ and WC appears to be a sensible solution to address these challenges. These carbides possess excellent wear resistance, hardness, and oxidation properties^4^. Research has demonstrated that combining Ni (Cr) with hard chromium carbides can enhance the toughness and oxidation resistance of the coating. Ni-based alloys are commonly employed in various industrial applications due to their high wear resistance under elevated contact pressures^20^. A clad powder based on 75Cr_3_C_2_ + 25(NiCr) is preferred to enhance the wear resistance properties of steel alloys and Ni-based superalloys at high temperatures^21^. The Cr_3_C_2_-NiCr cermet coating has gained wide industrial usage due to its excellent wear and corrosion resistance combination. Although the corrosion resistance is influenced more by porosity than the chemical composition, achieving a fully dense state and metallurgical bonding with the substrate is crucial^22^.

Despite the numerous studies conducted on laser and microwave clads, there is still a lack of available data regarding the material processing of clads developed using both laser and microwave energy under identical material conditions. This knowledge gap directly impacts the quality and engineering costs in a significant manner. To address this gap, current research uses laser and microwave energy to develop composite clads with a composition of 75 wt % Cr3C2 and 25 wt % NiCr on T91 steel. Comparative observations are reported on phase formation, microstructural characteristics, and Vickers microhardness, fretting/fatigue wear assessment of both types of clad developed.

## Experimental details

The material details, experimental procedure and characterization techniques used in the current work have been briefly discussed in the following sections.

### Substrate and powder materials

**Figure 1:**
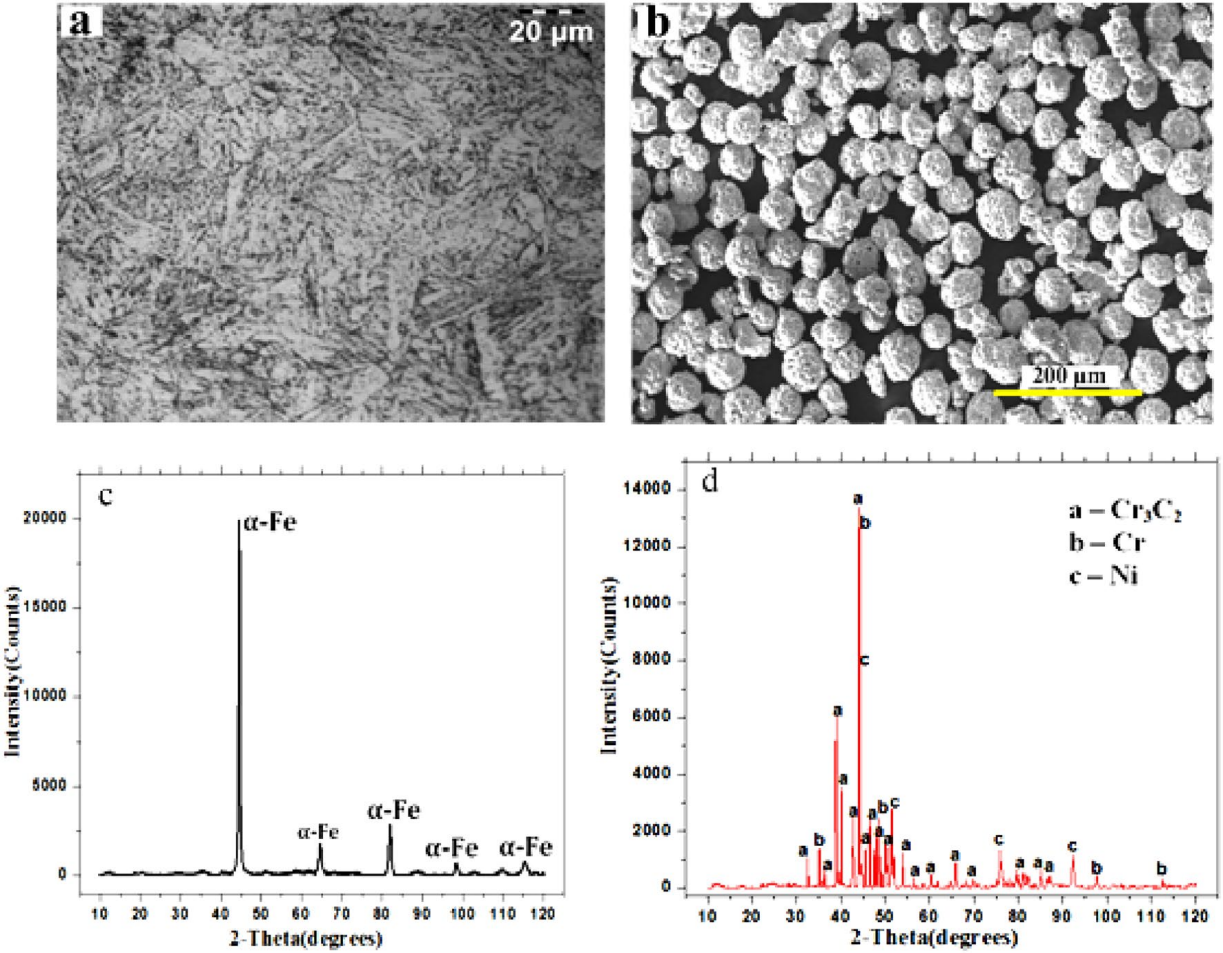
SEM images of (**a**) Substrate (T91) microstructure, (**b**) Cr_3_C_2_-NiCr powder morphology, (**c**) XRD spectrum of substrate T91, and (**d**) XRD spectrum of Cr_3_C_2_-25 NiCr powder.

In the current work, commercially available 75 wt% chromium carbide—25 wt% Nickel Chromium (75Cr_3_C_2_-25NiCr) composite powder (Make: Oerlikon metco (Woka 7202)) having a particle size of 45 µm was used to develop clads on T91 ferritic alloy steel. A microstructure of substrate T91 is shown in Fig. 1a. The particles of the clad powder had a spherical form. Figure 1b shows the typical shape of the unprocessed clad powder used for deposition. XRD pattern of Cr_3_C_2_-NiCr composite powder is illustrated in Fig. 1d. This shows the dominant presence of Cr_3_C_2_ along with NiCr. The Cr_3_C_2_ particles are liable for imparting higher hardness, while NiCr acts as a binder that offers greater matrix strength through its excellent adhesion properties and carbide wetting. The substrates were machined to the desired dimensions from the T91 steel plate. The chemical composition of chosen clad powder (Cr_3_C_2_-NiCr) and T91 substrate is presented in Table 1. The XRD spectrum of the substrate T91 shows the major dominance of ferrite iron Fig. 1c.

**Table 1:** Chemical composition (wt%) of the target materials.

Elements	Fe	Cr	Ni	Mo	C	Si
Substrate T91	Bal	8–9.5	0.04	0.85–1.05	0.07–0.14	0.2–0.5
Cr_3_C_2_-25NiCr	–	Bal	17.5–22.5	–	9.0–10.2	–

The clad powder and substrate preparation are also important in the development process. Therefore, the substrate was polished with a standard metallurgical process and cleaned with acetone before powder deposition. The clad powder was warmed at 200 °C for 24 h in a normal muffle furnace to eliminate possible moisture content. Cr_3_C_2_-NiCr-based composite clads are developed through two different processes described as follows.

### Laser cladding process

**Figure 2:**
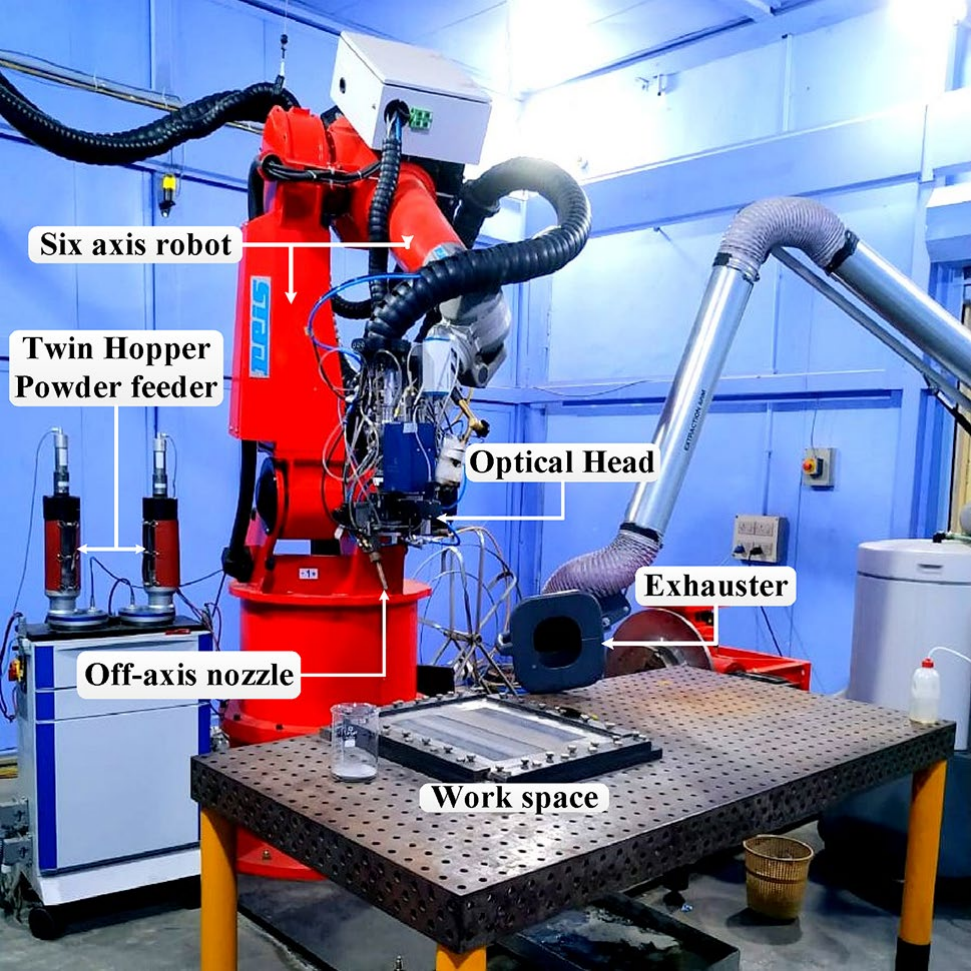
A diode laser experimental setup was used to develop Cr_3_C_2_-NiCr-based composite laser clads.

Cr_3_C_2_-NiCr-based composite clads were developed through laser energy on the T91 substrate. The laser cladding experimental setup equipped with a 10 kW diode laser consists of fiber delivery and an optic head system placed on a 6-axis robot with a square spot size of 6 mm. An off-axis powder-feeding nozzle assembly was employed to feed the powder on the substrate during laser interaction with argon gas. A vertical distance between the substrate and a laser beam was maintained by 14 mm. To optimize the process parameters, numerous experimental tests were carried out. Finally, laser clads were developed with 2000 W power by maintaining a 5 mm/s scanning speed with a powder feed rate of 8 g/min was retained. A Typical laser clad experimental system used is shown in Fig. 2.

### Microwave cladding process

**Figure 3:**
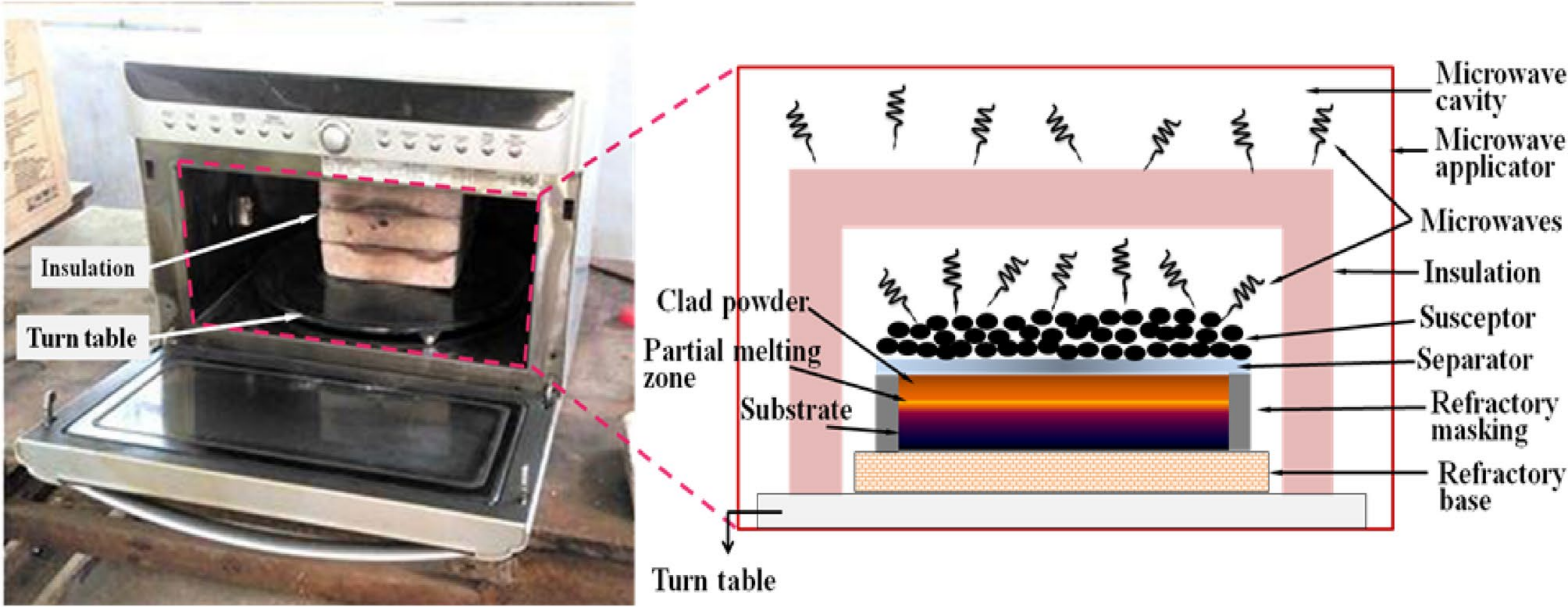
Microwave oven and schematic of hybrid heating setup.

In this process, Cr_3_C_2_-NiCr based clads developed through microwave energy on substrate T91. Before deposition of the clad powder, the flat T91 substrate was thoroughly cleaned using alcohol, ensuring its cleanliness. Initially, the clad powder was mixed with Araldite binder to prepare a slurry; the prepared slurry of clad powder was applied uniformly on a substrate with an approximate thickness of 1 mm. Experimental trials were carried out using a conventional microwave oven, with a 99% pure alumina plate (insulation), approximately 0.5 mm thick, kept on the slurry of clad powder applied on the substrate. The alumina plate performs as a separator between clad powder and the susceptor. The charcoal powder was used as a susceptor which initiates heating and helps to increase the temperature of the clad powder particles beyond its critical level. Once the clad powder reaches its critical temperature, these particles couple with an incident of microwave radiation, further leading to heated up and melting. The metallic substrate was placed on the refractory base. A highly microwave-absorbent material called a susceptor was employed to raise the temperature of the powder particles. Microwave hybrid heating was then used to melt the preplaced powder. Once the experimental configuration was ready, the arrangement of the hybrid heating setup was placed on the turn table and exposed to microwave radiation at the domestic microwave oven. The schematic of the experimental setup is shown in Fig. 3. Finally, microwave clads developed in a domestic microwave oven (Make: LG 28 L Charcoal Convection Microwave Oven, Model: MJ2886BFUM, Black). The microwave irradiation was carried out with a power of 900 W at 2.45 GHz. The process parameters have been optimized based on trial and error methods, similar to our previous work^3,16,23^. Further, more information on the development of clads via microwave hybrid heating (MHH) is reported elsewhere^12,24–26^.

### Characterization of clads

The Cr_3_C_2_-NiCr based clads developed through the above processes were sectioned across the thickness and were hot mounted in epoxy. The mounted samples were then polished using standard metallographic techniques. XRD phase analysis was carried out through a Rigaku diffractometer using Cu Kα X-ray at room temperature. The scan rate was 1°/min while the 10°–120° scan range was maintained. Microstructure and EDS studies were carried out through a field emission scanning electron microscope. Microhardness of the clads was carried out at 500 g load with 15 s dwell time through a Vickers' microhardness tester (VMHT Micro Hardness Tester). A consistent distance of 100 µm was used for all microhardness indentations.

### Wear test

The wear test was conducted using linear reciprocatory ball-on-plate tribometers (THT1000 and TRB3, Anton Paar, Austria, ASTM G133) to evaluate the wear behavior of rough polished wire-cut microwave-clad and laser-clad samples. The dimensions of the samples were 10 mm × 10 mm × 6 mm (length × width × height). Table 2 provides the details of the wear parameters employed during the test. To examine the fretting/fatigue wear behavior of both clad surfaces under different loading conditions, a static alumina ball indenter with a radius of 3 mm was employed. The indenter performed a linear reciprocating motion with a 4 mm amplitude. The worn clads were subjected to SEM analysis to investigate and identify the associated wear mechanisms after the wear test.

**Table 2:** Wear test parameters of fretting wear studies.

Load (N)	10	20
Sliding distance (m)	250	500
Temperature	Room temperature: 30 °C	
Frequency	4 Hz	
Amplitude of reciprocatory motion	4 mm	
Static partner	Alumina Ball (Dia: 3 mm)	

## Mechanism of clad development

**Figure 4:**
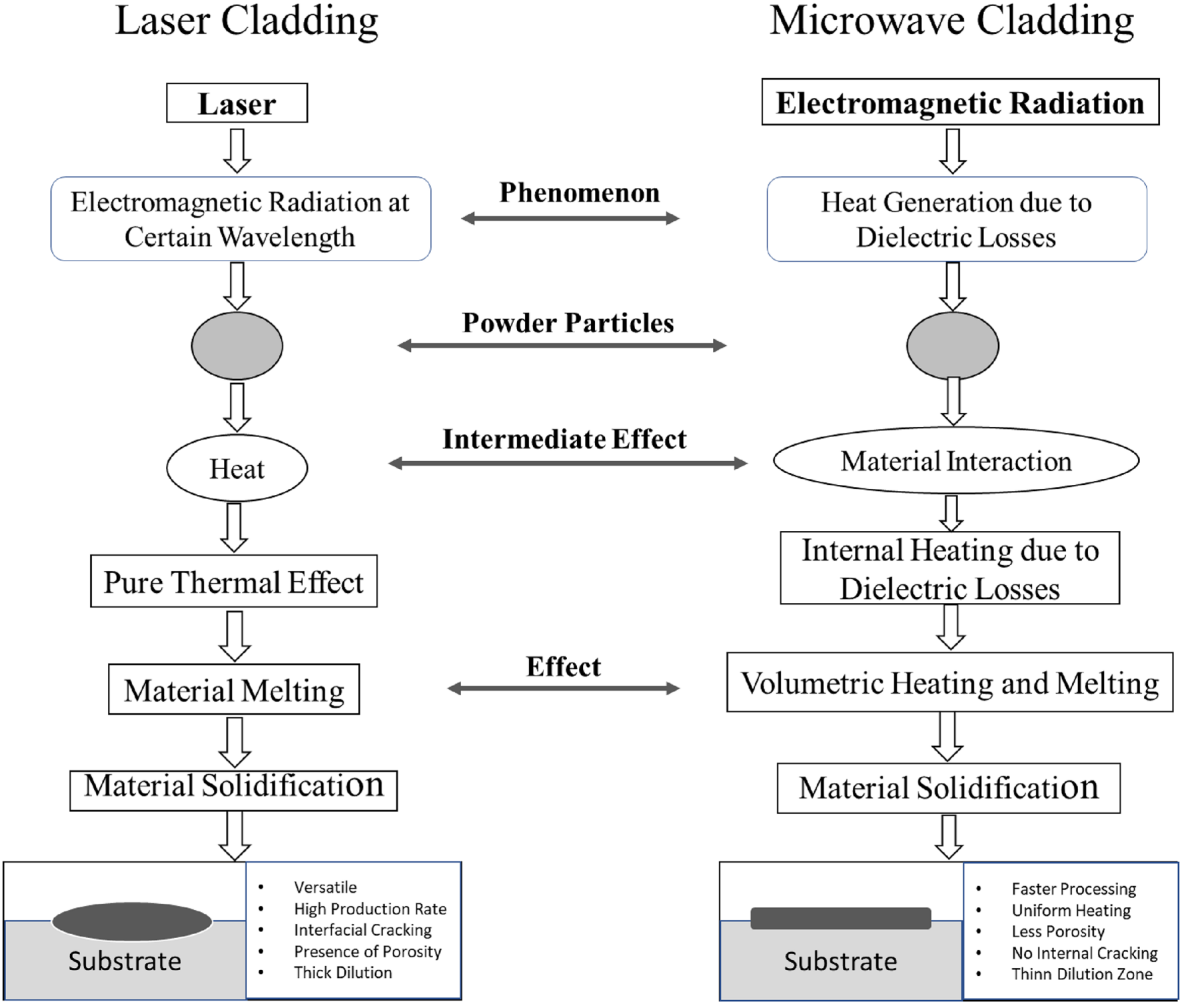
A schematic diagram shows single-particle processing through laser and microwave energy during clad development.

**Figure 5:**
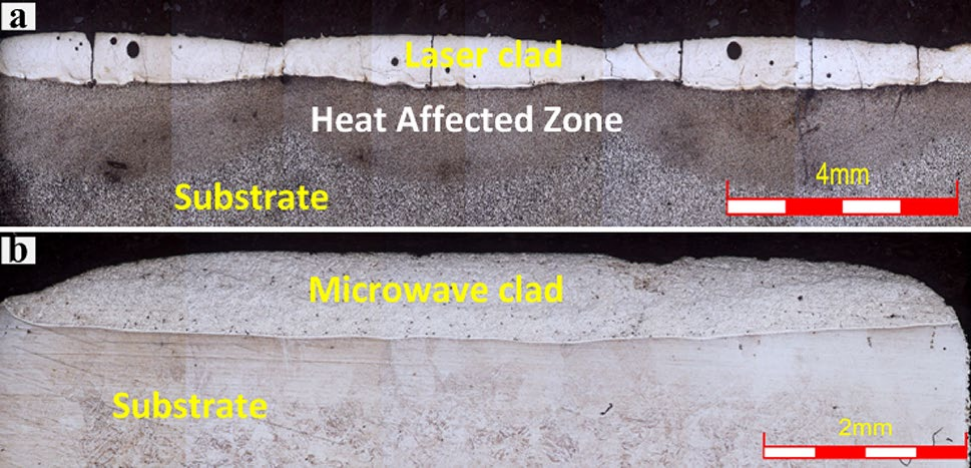
The typical optical image shows Cr_3_C_2_-NiCr-based area clads developed through, (**a**) Laser energy, (**b**) Microwave energy.

The modified surface behavior of engineering components across these two significant cladding methods can be understood well by attributing to their formation structure. The principles of surface development are illustrated schematically by a "single-particle processing" concept in Fig. 4. A laser cladding and sprayed powder produces a high-quality clad layer with minimal dilution. The powder particles are transported into the melt pool through a carrier gas and focussed at an angle of 38°–45° towards the target substrate. Complete melting and solidifying result in the dense microstructure. However, in the laser cladding process, the energy must be high enough to melt the powder particles and low to avoid the substrates' melting. The powder particles striking the substrate outside the melt pool bounce, but the particles striking the melt pool lead to melting completely. In laser clads, there are some concerns about residual stress development due to rapid solidification cracking, high thermal gradient and porosity^27^. Therefore, microcracks and porosity (Fig. 5a) cause spalling to the laser clads under severe working circumstances. In the microwave cladding process, heat is produced within powder particles due to dielectric losses, which further cause the volumetric nature of heating and subsequent melting. The molten clad powder particles further cause to raise the substrate temperature to its melting point and get fused (Fig. 4.). Upon solidification, a better-developed clad structure with uniform and dense microstructure, free from solidification cracking with negligible porosity can be seen (Fig. 5b).

## Results and discussion

Cr_3_C_2_-NiCr-based composite clads are developed through laser and microwave energy and are characterized through various techniques, and the findings are discussed in the subsequent sections.

### XRD phase analysis

**Figure 6:**
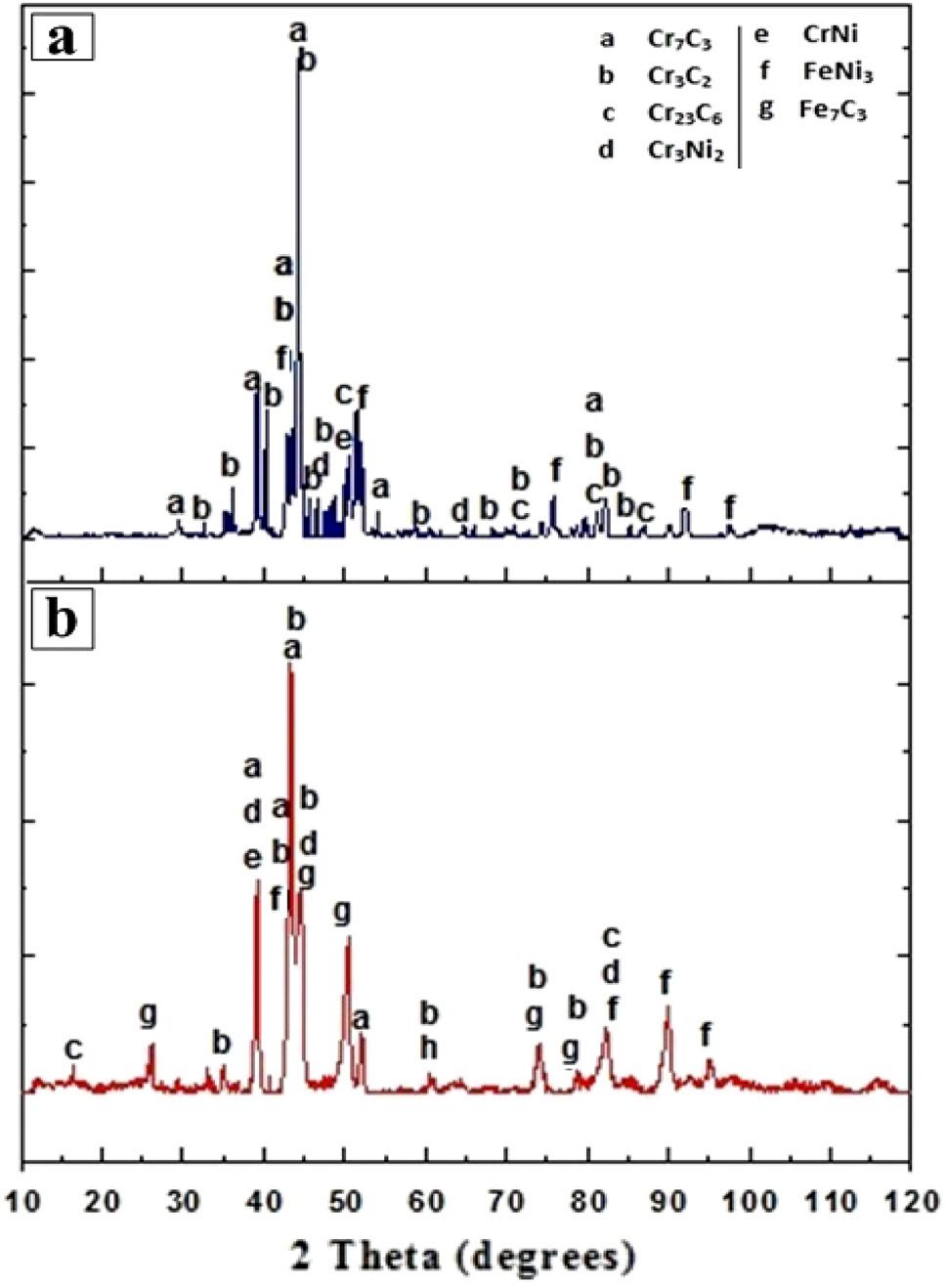
A typical XRD spectrum of Cr_3_C_2_-NiCr based clads of (**a**) laser energy, (**b**) Microwave energy.

An XRD spectrum of clads developed through laser energy and microwave energy is shown in Fig. 6. The clad spectrum of both the process evidenced the common presence of phases like chromium carbides (Cr_7_C_3_ Cr_3_C_2_, Cr_23_C_6_), Iron Nickel (FeNi_3_) and chromium-nickel (Cr_3_Ni_2_, CrNi). However, these iron carbides (Fe_7_C_3_) phases are observed in clads developed through microwave energy. XRD spectrum of laser clad surface (Fig. 6a) reveals that most peaks are chromium carbides, and minor peaks like iron-nickel and chromium-nickel are observed. It is clear that the decarburization of Cr_3_C_2_ results in the formation of chromium carbides such as Cr_7_C_3_ and Cr_23_C_6_. Cr_7_C_3_ is primarily formed from the decarburization of Cr_3_C_2_ due to the massive melting state of heating at the laser cladding process. This is confirmed by the fact that many Cr_7_C_3_ are found around the Cr_3_C_2_ particles^28^. Thus, a proportion of carbon is ideally precipitated as Cr_23_C_6_. The high-temperature melting of the laser cladding process leads to cause the partial dissolution of the primary Cr_3_C_2_, and this might be one of the major possibilities for the formation of types of chromium carbides. Such behavior enhances the carbon and chromium content of the melt pool, which stimulates the formation of many other carbide phases during most of the non-equilibrium cooling process. The formation of various chromium carbides (Cr_3_C_2_ and Cr_23_C_6_) has also been recorded earlier for the laser cladding of Ni60-Cr_3_C_2_^10^. Minor peaks such as chromium-nickel (Cr_3_Ni_2_, CrNi) are formed by the NiCr binder.

This NiCr binder will likely melt initially and crystallizes some chromium carbide in a liquid phase (Cr_3_Ni_2_) that may be rich in Cr, and C. Iron-nickel (FeNi_3_) may be due to diffusion of iron elements from the target surface to the clad, which is a clear proof of metallurgical bonding of substrate to clad. Another interesting observation seems to be the development of ferromagnetic FeNi_3_ intermetallic, even though chosen clad powder was iron-free (Table 1). These results indicate the dilution of elements in which iron has been diluted from the substrate. The formation of this type of intermetallic was also reported earlier^12^. The typical XRD spectra of composite clad (Cr_3_C_2_-NiCr) developed through microwave energy are shown in Fig. 6b. The existence of different phases such as chromium carbides (Cr_7_C_3,_ Cr_3_C_2_, Cr_23_C_6_), iron-nickel (FeNi_3_) and chromium-nickel (Cr_3_Ni_2_, CrNi), iron carbide (Fe_7_C_3_) can be seen in XRD test. The decarburization of Cr_3_C_2_ particles during microwave hybrid heating forms Cr_7_C_3_ and Cr_23_C_6_. Cr3Ni2 and FeNi_3_ phases might be due to the diffusion of chromium, nickel and iron elements from the substrate to clad at elevated temperature, which is a clear indication for metallurgical bonding of substrate to clad. The iron carbides phase (Fe_7_C_3_) might be attributed to the dilution of iron elements from the substrate to the clad region during the microwave cladding. These phases were not noticed on the laser-clad surface, possibly due to the rapid solidification and diffusion rate being less than the microwave cladding process. As discussed in the EDS analysis, the clad powder Cr_3_C_2_-NiCr was observed to be completely intermixed and fused within the substrate. The developed clad surface must be properly mixed with the base material. Therefore diffusion rate of the substrate is unavoidable. The higher diffusion rate of microwave clad results in the gradual interaction between the substrate and clad powder, further forming the iron carbides. The formation of these iron carbides indicates the cause of excellent metallurgical bonding during hybrid microwave heating. Finally, it is observed from both processes that there is a good amount of chromium carbides segregated on the developed clad layer along with intermetallic, which further helps to increase the hardness and wear resistance of the developed coatings of both processes.

### Microstructural observation

The microstructural study supports understanding the different phases present, their composition, grain boundary, inclusion, porosity, etc., appearing in the substance under examination. It helps to examine the microstructure's influence on clads' different properties. As a result, studies have been conducted on the microstructures of the developed clad.

**Figure 7:**
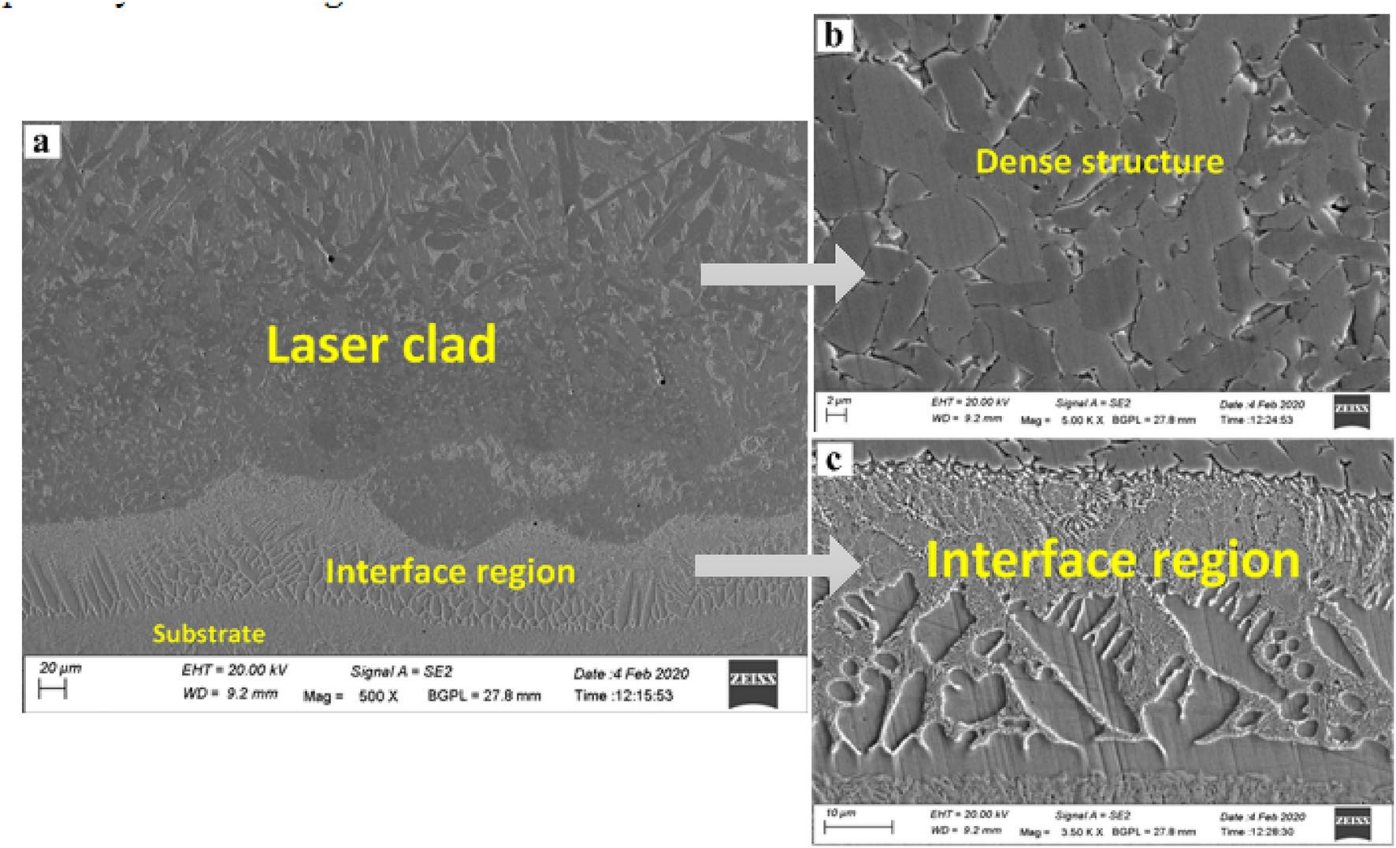
Microstructure of Cr_3_C_2_-NiCr based laser clad cross-section: (**a**) a typical FE-SEM image of a transverse section of Cr_3_C_2_-NiCr laser clad, (**b**) a Magnified view of the dense laser clad structure, (**c**) Magnified view of the interface region.

The microstructures of the Cr_3_C_2_-NiCr-based composite laser clad are shown in Fig. 7. The structure is completely dense interdendritic with a nickel-rich alloy phase and dendrites with chromium carbide spaces. The developed microstructure is typically solidified, with carbides as dendrites and a rich Ni phase dominating interdendritic spaces. It is also noticed that various columnar dendrites are growing perpendicular to the interface layer and interdendritic structure in the bottom part of the carbide layer, and few dendrites are noticed in the intermediate part of the clad layer. It is reported that the characteristics of this type of typical structure are directly related to the solidification rate (R) and temperature gradient (G) of the liquid alloy in the laser melt pool. At the beginning of solidification, there was a larger G value and a small R-value in the bottom part of the clad layer. This value of G/R gradually reduced to zero closer to the surface with the solidification process, which further leads to the cause for the above crystal growth^29^. Some coarse columnar dendrites were replaced by tiny dendrites covered by a bright eutectic. This was caused by the extremely high melting point of Cr_3_C_2_ particles, which were abundantly present in the melted pool and would alter the temperature fields before the liquid–solid boundaries, affecting the solidification structure (Fig. 7c). The absence of microcracks and porosity has shown that the technical parameters considered for this study have ensured a high quality of the laser cladding process. A similar thick planar crystal zone between bonding and HAZ was also reported elsewhere^30^.

**Figure 8:**
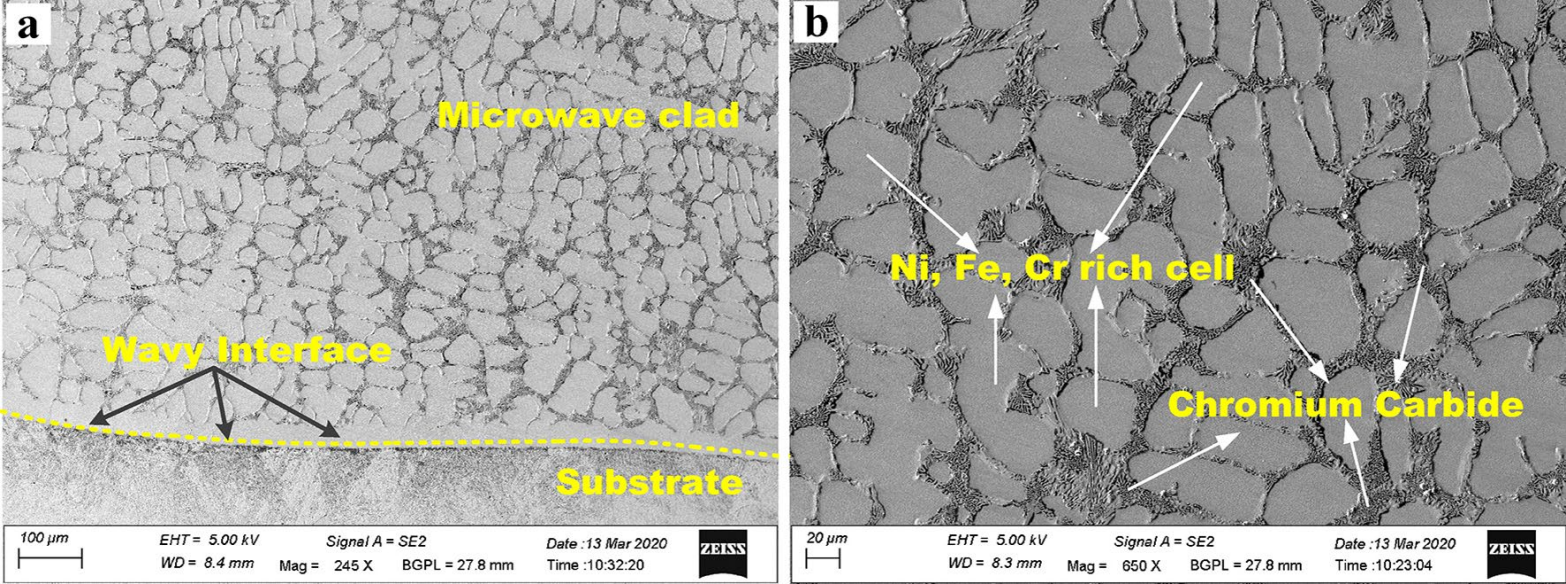
Microstructure of Cr_3_C_2_-NiCr based microwave clad cross-section: (**a**) a typical FE-SEM image of a transverse section of Cr_3_C_2_-NiCr microwave clad, (**b**) Magnified view of the microwave clad structure.

A typical cross-section of the developed microwave-clad cross-section is shown in Fig. 8. The microwave cladding process offers precise control over the heating parameters, such as power level and heating time, allowing for optimization of the cladding process. This control enables the formation of a desirable microstructure and facilitates the elimination of porosity, resulting in a pore-free structure with enhanced bonding strength. Compared to other cladding techniques, such as laser or thermal spraying, microwave cladding can achieve better metallurgical bonding. The unique characteristics of volumetric heating, rapid heating and cooling rates, and precise control over heating parameters contribute to the superior performance of the microwave clad, making it stand out among alternative cladding methods. It is observed that the developed microwave-clad shows good bonding with the substrate by partial mutual diffusion of elements. A substrate-clad interface is free from any noticeable discontinuities. The observed wavy interface between the substrate and clad structure can be seen (Fig. 8a) due to localized melt pool current during microwave heating. The melting rate of clad powder and substrate directly depends on the melt pool current. It was also noticed that the developed microwave clads are free from observable pores, and interfacial cracks and clad regions appear defect-free. The magnified view of the clad section is shown in Fig. 8b, which shows that hard chromium carbide particles remain consistently dispersed within the soft nickel matrix. The nickel particles of the clad powder start melting first as microwaves initiate interaction during microwave heating, and hard carbide particles continue to be evenly distributed within the soft matrix Fig. 8b. The defect-free clad structure can be noticed due to the melt pool's slower solidifying rate. Various carbides, initially chromium carbide and other complex metallic carbides, are partly agglomerated due to the melt pool current and remain consistently disseminated. These carbides could further strengthen the developed clad structure and act as strengthening in the developed composite. The nature of the volumetric heating character is directly associated with hybrid microwave heating, which is affected by a minimal thermal grade in the exposed surface of the microwave. The carbides are distributed uniformly in the clad structure, which may result from the melt pool's slow solidification rate. Similar types of metallic carbides uniformly distributed are reported elsewhere^17,31^. The formation of cellular dendrites was not noticed anywhere in the developed microwave-clad structure. This could be due to a uniform thermal gradient that does not allow the cell to transition into dendrites^32^.

### EDS analysis

**Figure 9:**
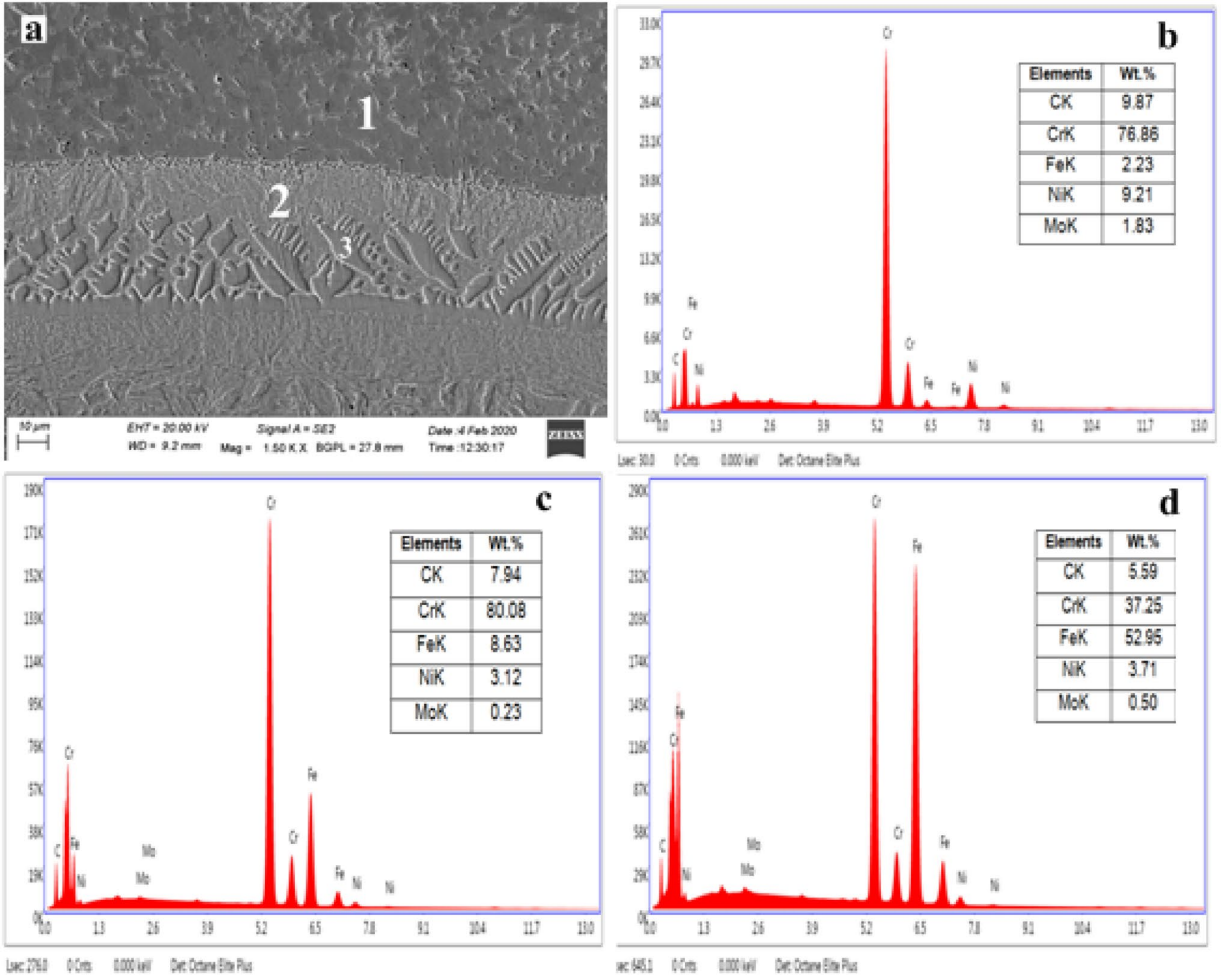
Typical EDS studies of the laser clad: (**a**) FE-SEM picture showing locations (EDS studies), (**b**) EDS studies of location (point 1) on developed clad, (**c**) EDS spectrum on the interface (point 2), (**d**) EDS studies of grey phase (point 3).

**Figure 10:**
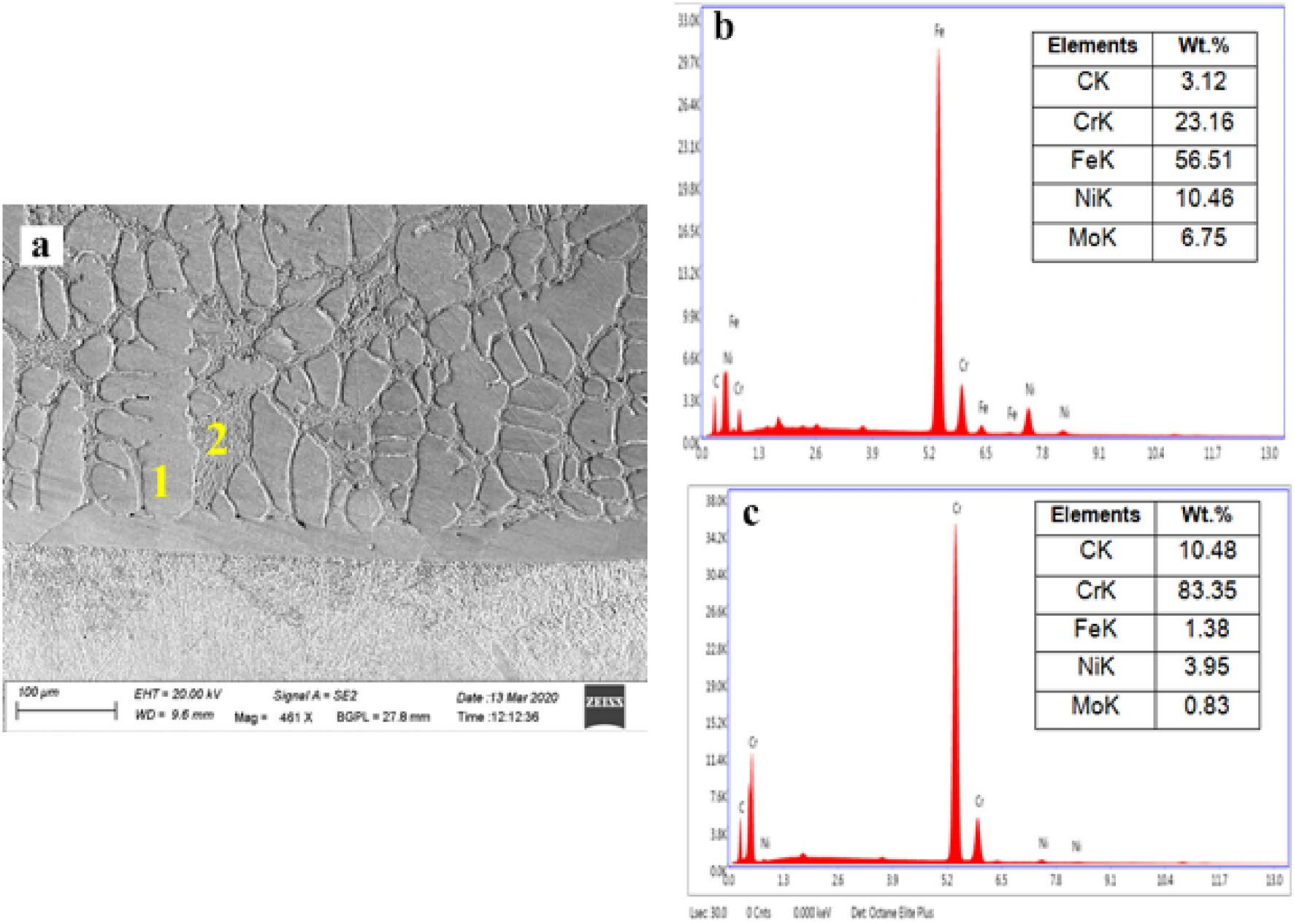
Typical EDS spectrum of the microwave clads (**a**) FE-SEM image revealing locations of EDS studies, (**b**) EDS studies of a location on clad (point 1), (**c**) EDS studies on grain boundary of the clad matrix (point 2).

An EDS analysis was conducted at various locations, and equivalent results are reported in Figs. 9 and 10 correspondingly. As observed from the microstructure of the laser clad shown in Fig. 9a, three phases in the microstructure can be seen – grey, light white and light grey, mentioned as 1, 2 and 3, correspondingly. Point number 1 exhibits metallic carbides of the developed clad matrix, whereas points 2 and 3 designate the positions in the interface region. EDS studies of the first point (Fig. 9b) reveal that the occurrence of Cr and C influences the grey phase with impacts of approximately 76.86% and 9.87%, respectively. The existence of such hard metallic carbides (Cr_x_C_Y_) in the laser clads, as discussed in section "XRD phase analysis", designates the prospects of showing better wear resistance.

Meanwhile, the EDS study of point 2 (white phase) on the interface region denotes the existence of elements such as Cr, C and Fe (Fig. 9c). Point number 3 of the EDS study (light grey phase) of the interface, the region indicates the existence of the major elements such as Fe, Cr, C and Ni (Fig. 9d). A molten clad powder's clad layer can cause dilution of Fe and Mo, thus resulting in their presence. Thus, the clad comprises a relatively strong matrix (Fe–Cr–Ni based). Therefore, the uniform distribution of the carbides acts as reinforcements, which further helps to increase the wear resistance of tough metallic matrixes of the Cr_3_C_2_-NiCr-based composite laser clad. It can be observed that the fine dendritic structure of the interface region helps to improve the tribological properties. Earlier research has demonstrated that this kind of structure enhances the tribological properties of the target component. The major reason for this incidence is expected to be the dilution of Fe elements of clad powder and metallurgical bonding. Fe content was found in remelted coatings based on EDS analysis^33^.

As evidenced by the developed clad microstructure (Fig. 10a), two unique phases are observed—grain and the grain boundary, which showed as 1 and 2, respectively. Point number 1 signifies the grain and the developed clad matrix influenced by the occurrence of Fe, Ni and Cr with distributions of roughly 56%, 10%, and 23%, respectively, as shown in Fig. 10b. It is observed that a higher percentage of iron is attributed to the melting pool of molten clad powder caused by localized convective currents, which further leads to elemental interaction between clad powder and the target substrate. Which clears, the developed clad comprises a tough matrix (Fe–Ni–Cr). Further, Fe, Ni, and Cr had formed the intermetallics such as Cr_3_Ni_2_, FeNi_3_ is observed in the XRD studies of microwave clad (Fig. 6b). The EDS studies of point 2 (Fig. 10c) marked on grain boundary enriched with Cr and C contribute roughly 83% and 10%, correspondingly. This indicates that the grain boundary of the developed clad has metallic carbides reinforced in the developed clad matrix. The occurrence of metallic carbides in developed microwave clad signifies the probability of performing superior hardness and resistance to erosion. Therefore, it is a clear sign from the EDS analysis that the uniform distribution of hard metallic carbides (Cr_3_C_2_, Cr_7_C_3_, Cr_23_C_6_) acts as reinforcement in the tough matrix of microwave-clad (Cr_3_C_2_-NiCr), which are expected to provide resistance to wear at elevated temperature.

### Microhardness of clads

**Figure 11:**
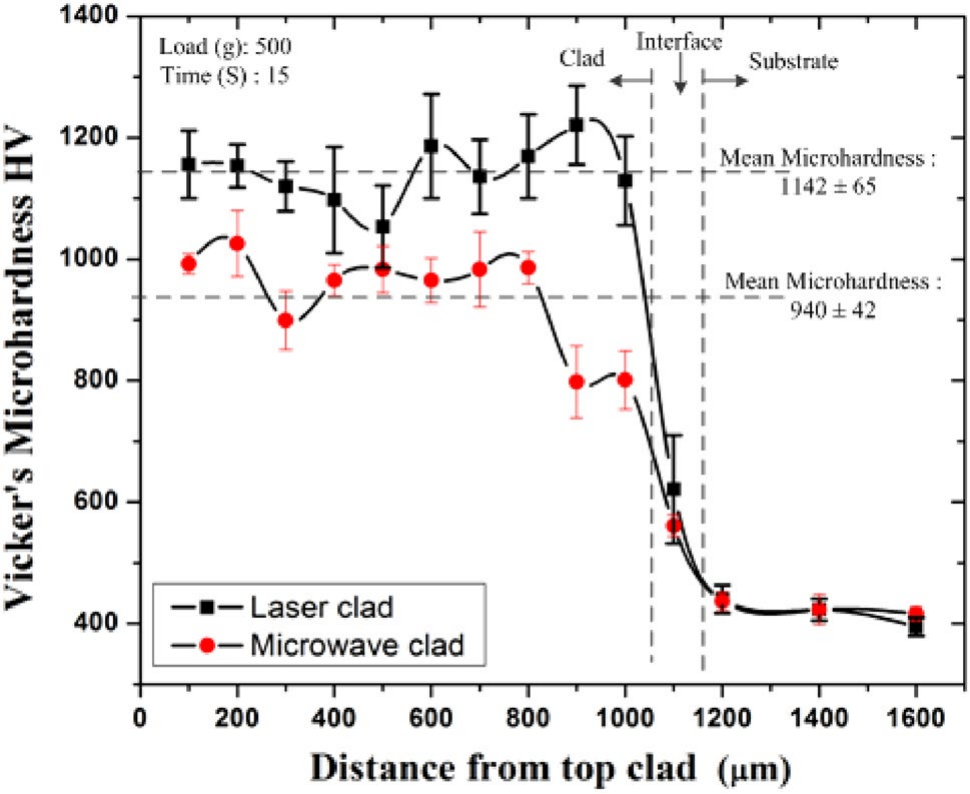
Shows Vickers’s microhardness profile of Laser clad (black) and Microwave clad (red).

The microhardness was measured across the cross-sections of laser and microwave clad. It was pursued to know the microhardness variation across the developed clad layer and the base substrate. The microhardness distributions are illustrated in Fig. 11. The average microhardness of the substrate (T91) was 418 ± 12HV. However, the authors observed that the microwave-clad had hard chromium carbide particles consistently dispersed within the soft nickel matrix. The X-ray phase analysis of both the processes evidenced the common presence of phases like chromium carbides (Cr_7_C_3_, Cr_3_C_2_, Cr_23_C_6_), Iron Nickel (FeNi_3_) and chromium-nickel (Cr_3_Ni_2_, CrNi), despite these phases iron carbides (Fe_7_C_3_) are observed in the developed microwave clads. The homogeneous distributions of such carbides in the developed clad structure of both processes indicated higher hardness. The typical microhardness of the laser-clad (1142 ± 65HV) was about 22% higher than the microwave-clad (940 ± 42 HV). As discussed in the EDS analysis, the clad powder Cr_3_C_2_-NiCr was completely intermixed and fused within the substrate (section "Microstructural observation".). Diffusion from the substrate is unavoidable because the developed clad surface must be mixed properly with the base material. Therefore, minor variations can be noticed in both clads' hardness profiles due to the Fe element from the target substrate. However, various waviness zones were seen in microhardness values across the sections; these non-uniform distributions across the segment leads to attributed to the alteration in hardness of the tough metallic matrix and hard carbide-based reinforcement, as well heating effects of both the process resulting in microstructural changes caused by successive clads developed through laser and microwave energy. It was reported experimentally that whenever hard carbide particles were fused into a softer surface, the hardness of the same surface was improved^34,35^. Many other scientists have also experienced similar behavior in the fusion of hard particles into softer surfaces, and the results were consistent with the findings of Li Pengting et al.^36^. It is also observed that the microhardness of laser clad is more, possibly due to the formation of a dendrite structure that limited the plastic distortion formed by the indenter. Hence, the developed laser-clad surface was thus strengthened by the dendrite structure. The residual stresses can also affect the microhardness of the developed clad. The laser cladding process often generates higher transient temperatures and thermal gradients, which can induce higher residual stresses. These compressive residual stresses can increase the microhardness of the laser clads^37,38^. The laser cladding process typically implies a faster cooling rate than the microwave cladding process. Laser cladding often involves faster cooling and rapid solidification, leading to fine and evenly distributed microstructures, such as fine dendritic structure, which is generally associated with higher microhardness^39,40^. In the Microwave cladding process, with its unique heating process, a relatively slower cooling rate with different solidification behavior may result in different residual stress profiles, potentially leading to lower microhardness than the developed laser clads. However, The Cr_3_C_2_-NiCr based clads developed through both the process had a much higher microhardness than the target substrate, which may be primarily related to the formation of carbides (Cr_7_C_3_, Cr_3_C_2_, Cr_23_C_6,_ Fe_7_C_3_).

### Wear behavior of laser and microwave clads

The wear characteristics of the microwave-clad and laser-clad samples were evaluated through a linear reciprocatory ball-on-plate wear test. The test parameters, including load variations and sliding distance, are presented in Table 3. The investigation focused on the fretting/fatigue wear behavior of the samples. During the initial test conditions, it was observed that the microwave-clad samples exhibited a coefficient of friction (COF) of 0.80 µ compared to the COF of laser-clad samples of 0.61 µ. This can be attributed to the lower hardness of the developed microwave clads.

**Table 3:** Coefficient of friction (COF) observations of wear test results.

Load (N)	10	20
Sliding distance (m)	250	500
Microwave clad COF(µ)	0.80	0.84
Laser-clad COF (µ)	0.61	0.58

**Figure 12:**
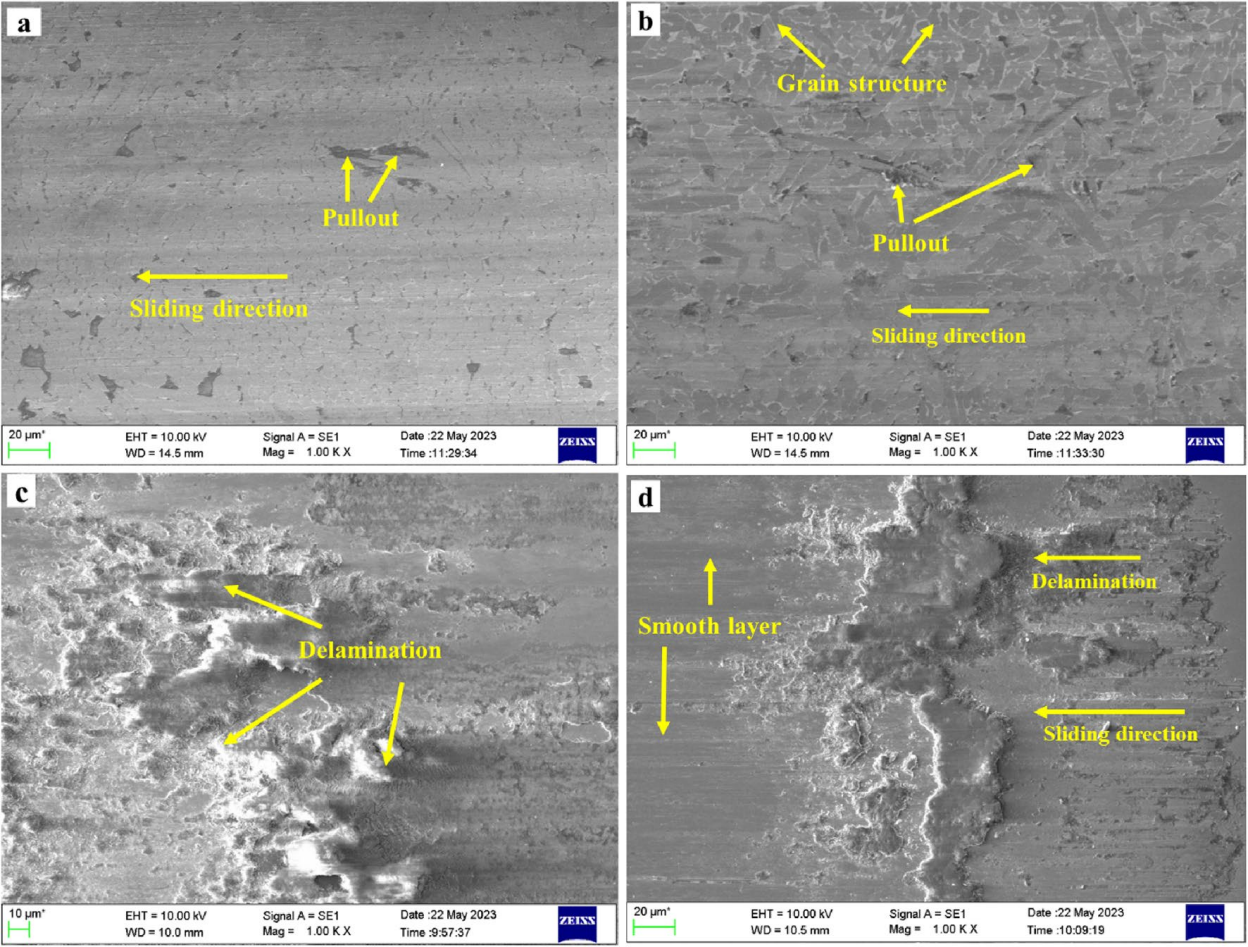
Worn surface morphologies of (Cr_3_C_2_-NiCr) based cladding: Laser clads (**a**) 10 N, 250 m (**b**) 20 N, 500 m; Microwave clads (**c**)10 N, 250 m, (**d**) 20 N, 500 m.

However, as the test parameters increased, the COF of the microwave-clad samples drastically increased due to the tearing of the developed surface layer, as illustrated in Fig. 12c,d. On the other hand, the laser-clad samples demonstrated a lower COF as the load and sliding distance increased, as depicted in Fig. 12a,b. This suggests that chromium carbides in the laser clads acted as an internal lubricant, reducing friction coefficient and improving wear resistance. The comparison between microwave and laser clads regarding wear behavior revealed the different characteristics and performance of the two techniques. While microwave clads initially exhibited a lower COF, tearing the surface layer resulted in increased friction. In contrast, laser clad demonstrated a consistently lower COF, indicating its superior lubricating and wear-resistant properties attributed to chromium carbides.

Figure 12a,b presents the worn-out images of the laser-clad samples. In contrast to the microwave-clad samples, the laser clad exhibited superior wear resistance under the test conditions. Hard carbide elements such as Cr_7_C_3_ and Cr_23_C_6_ played a significant role in preventing the detachment of melted particles from the surface, enhancing the resistance to wear. The uniform and robust surface of the laser clad resulted in minimal material loss during testing. The incorporation of carbides in the dendritic region increased hardness and wear resistance, albeit reducing the mean free path^40,41^. Figure 12c,d showcases the worn surface of the microwave-clad samples, which exhibited more surface damage and material loss than the laser-clad samples. Microcutting of the relatively soft binder, followed by carbide loosening and pullout, contributed to the material removal. Furthermore, at higher loads, fatigue-induced carbide fracture led to material loss. The main wear mechanisms observed during fretting wear were matrix flaking, carbide fracture, and pullout, possibly due to hard chromium carbide particles consistently dispersed within the soft nickel matrix.

The comparison between laser and microwave clads, as depicted in the worn-out images in Fig. 12, highlights the differences in wear behavior. Laser clads demonstrated greater wear resistance due to the effective retention of melted particles and a uniform and durable surface. On the contrary, microwave clads demonstrated greater surface damage and material loss, predominantly attributed to micro-cutting, carbide loosening, fatigue-induced carbide fracture, and the formation of a smooth layer. Figure 12d illustrates the development of a smooth layer during fretting wear, which entails the creation of a polished or relatively flat surface on the microwave clad under fretting conditions. This smooth layer is typically observed in the region of the worn surface and can be attributed to multiple factors. During fretting wear, the cyclic loading and relative motion between two contacting surfaces result in repetitive micro-slip and sliding at the interface. This motion leads to the removal of surface irregularities on the clad and the formation of wear debris. As the fretting process persists, the initial roughness and irregularities on the microwave-clad surface gradually diminish, resulting in a smoother surface appearance. Various factors, including material properties, contact conditions, and lubrication, can influence the formation of a smooth layer. In certain instances, protective films or oxide layers on the material surface can contribute to developing a smooth layer by acting as a barrier against further surface damage or wear^42^. These findings provide valuable insights into the wear mechanisms associated with laser and microwave cladding, emphasizing the advantages of laser cladding in terms of wear resistance and material preservation.

## Conclusion

The current work institutes the probability of using microwave energy equal to the laser energy to develop Cr_3_C_2_-NiCr-based composite clads on the T91 steel substrate. Major observations are drawn from the current work as follows.

- Cr_3_C_2_-NiCr-based composite clads have been developed on the T91 steel substrate using laser and microwave irradiation. Both processes developed clads that showed excellent metallurgical bonding with the target substrate.
- Cr_3_C_2_-NiCr-based composite laser clads dense interdendritic structures with nickel-rich phases and dendrites with chromium carbide spaces. In the case of microwave clads, the hard chromium carbide particles consistently dispersed within the soft nickel matrix.
- The clad spectrum of both the process evidenced the common presence of phases like chromium carbides (Cr_7_C_3,_ Cr_3_C_2_, Cr_23_C_6_), Iron Nickel (FeNi_3_) and chromium-nickel (Cr_3_Ni_2_, CrNi); despite these phases, iron carbides (Fe_7_C_3_) are observed in the clads developed through microwave energy.
- The Cr_3_C_2_-NiCr-based composite clads developed through both processes had a much higher microhardness than the target substrate, which may be primarily related to the formation of carbides (Cr_7_C_3_, Cr_3_C_2_, Cr_23_C_6,_ Fe_7_C_3_).
- The average microhardness of the developed clads of laser and microwave energy increased by 2.7 times and 2.3 times compared to the substrate's average microhardness (418 ± 18HV).
- The microwave cladding process is a cost-effective, eco-friendly, and energy-efficient material processing method.
- The study analyzed the wear behavior of microwave and laser-cladding samples using a linear reciprocatory ball-on-plate test. Microwave-clad samples had a slightly lower coefficient of friction but increased as load and sliding distance increased. Laser cladding showed a lower coefficient, possibly due to chromium carbide's internal lubrication.
- The laser-cladding samples showed superior wear resistance due to hard carbide elements, preventing melted particles from detaching and resulting in a uniform, durable surface. Microwave-clad samples showed more surface damage and material loss, driven by micro-cutting, carbide loosening, and fatigue-induced carbide fracture.

## Data Availability

The datasets used and analyzed during the current study are available from the corresponding author upon reasonable request.
